# A flexible design for advanced Phase I/II clinical trials with continuous efficacy endpoints

**DOI:** 10.1002/bimj.201800313

**Published:** 2019-07-12

**Authors:** Pavel Mozgunov, Thomas Jaki

**Affiliations:** ^1^ Medical and Pharmaceutical Statistics Research Unit Department of Mathematics and Statistics Lancaster University Lancaster UK

**Keywords:** combination trial, continuous endpoint, nonmonotonic efficacy, Phase I/II clinical trial

## Abstract

There is growing interest in integrated Phase I/II oncology clinical trials involving molecularly targeted agents (MTA). One of the main challenges of these trials are nontrivial dose–efficacy relationships and administration of MTAs in combination with other agents. While some designs were recently proposed for such Phase I/II trials, the majority of them consider the case of binary toxicity and efficacy endpoints only. At the same time, a continuous efficacy endpoint can carry more information about the agent's mechanism of action, but corresponding designs have received very limited attention in the literature. In this work, an extension of a recently developed information‐theoretic design for the case of a continuous efficacy endpoint is proposed. The design transforms the continuous outcome using the logistic transformation and uses an information–theoretic argument to govern selection during the trial. The performance of the design is investigated in settings of single‐agent and dual‐agent trials. It is found that the novel design leads to substantial improvements in operating characteristics compared to a model‐based alternative under scenarios with nonmonotonic dose/combination–efficacy relationships. The robustness of the design to missing/delayed efficacy responses and to the correlation in toxicity and efficacy endpoints is also investigated.

## 1 INTRODUCTION

There is growing interest in oncology in integrated Phase I/II clinical trials evaluating both toxicity and efficacy of a novel agent simultaneously (Yin, 2013). Compared to separate Phase I and Phase II clinical trials, a Phase I/II study allows for acceleration of the drug development and for the reduction of its costs (Wages & Conaway, 2014). The integrated trials are of particular interest when a molecularly targeted agent (MTA) is studied as the dose–efficacy relationship for MTAs can exhibit either a plateau or umbrella shape (Riviere, Yuan, Jourdan, Dubois & Zohar, 2016; Wages & Tait, 2015). At the same time, the dose–toxicity relationship might be nondecreasing. Therefore, the toxicity cannot be considered as a proxy for efficacy as usually assumed in many Phase I clinical trials. In this case, considering the toxicity endpoint alone might not provide sufficient information about the dosing regimen to be recommended for further phases. The goal of Phase I/II single‐agent clinical trial is to find the optimal biologic dose. There are different definitions of the optimal biologic dose which depend on the context of the trial (Mozgunov & Jaki, 2019). In this work, we will call a dose optimal if it corresponds to a toxicity probability below the upper toxicity bound ϕ (the dose is safe) and to the highest efficacy above a lower efficacy bound ψ (the dose is efficacious).

Recently, several Phase I/II designs for clinical trials involving MTAs were proposed in the literature (see e.g., Cai, Yuan & Ji, 2014; Mozgunov & Jaki, 2019; Riviere, Yuan, Jourdan, Dubois & Zohar, 2016; Wages & Tait, 2015). These designs, however, are only applicable to trials with both toxicity and efficacy outcomes being binary. At the same time, continuous endpoints can potentially carry more information about the agent's profile (Ivanova & Kim, 2009; Wang & Ivanova, 2015). Therefore, it becomes more common to plan trials with continuous efficacy and binary toxicity endpoints. Historically, one of the first designs for Phase I/II trials of a single agent with continuous efficacy was proposed by Bekele and Shen (2005). While being able to account for the correlation between toxicity and efficacy endpoints, this design assumes monotonically increasing dose–toxicity and dose–efficacy relationships. This was found to limit the ability of the design to find the “optimal” dose in scenarios with a plateau in the efficacy curve (Mozgunov, Jaki & Paoletti, 2018b). As an alternative, Hirakawa (2012) and Yeung et al. (2015, 2017) proposed designs for continuous efficacy endpoints that model the plateau in the dose–efficacy relationship. Specifically, Hirakawa (2012) proposed a design motivated by a squamous cell carcinoma Phase I trial. The squamous cell carcinoma antigen (SCCA) was used as a tumour marker in patients with cervical carcinoma. For each patient, the SCCA level was measured at baseline (i.e., prior to receiving treatment) and on day one of the second cycle of a therapy, and the change in the SCCA levels (on the logarithmic scale) was used as the continuous efficacy outcome in this study.

The majority of designs for continuous efficacy endpoints were developed for single‐agent studies, but it is a common practice to combine an MTA with standard therapy or with another MTA to improve its therapeutic index (Sharma & Allison, 2015). Nevertheless, Phase I/II designs for combination or dose‐schedule trials have received very limited attention in the literature due to its complexity. Hirakawa (2012) used the setting of the SCCA trial to propose a design for the combination trials which, however, requires seven and four parameters to be specified for the combination–efficacy and combination–toxicity relationships, respectively. Consequently, it can be challenging to extend it to trials with complex treatment–toxicity and treatment–efficacy relationships (as e.g., investigated by Mozgunov & Jaki, 2019) due to the limited sample size.

The aim of this paper is to extend the flexible Phase I/II design by Mozgunov and Jaki (2019) that was originally proposed for trials with binary toxicity and efficacy endpoints to the case of a continuous efficacy endpoint. The design uses no monotonicity or parametric assumptions about toxicity/efficacy relationships and can be applied to advanced clinical trials with complex toxicity and efficacy structures. The continuous outcomes are incorporated in the escalation criterion by the transformation of the efficacy endpoint to the unit interval as conventionally applied in benefit–risk analysis (Saint‐Hilary, Cadour, Robert & Gasparini, 2017; Saint‐Hilary, Robert, Gasparini, Jaki & Mozgunov, 2018). The choice of transformation is studied in detail. We study the performance of the novel design in a comprehensive simulation study and compare it to the performance of currently used methods.

The rest of the paper proceeds as follow. The selection criterion and the proposed transportation for a continuous endpoint are introduced in Section 2. The design and safety and futility constraints are provided in Section 3. A comparison to alternative methods in the context of single‐agent and dual‐agent combinations is given in Section 4 and a sensitivity analysis provided in Section 5. Section 6 concludes with a discussion.

## 2 METHODS

The proposed design is flexible and can be applied to many clinical trials, for examples, single‐agent, combination, dose‐schedule or combination‐schedule trials. We use the term “regimen” as a generic name for the objects of study in the Phase I/II clinical trial. Depending on the context, the regimen can be dose, combination, dose‐schedule, combination‐schedule, and so forth. We then say that the goal of Phase I/II clinical trial is to find the optimal biological regimen (OBR) among *m* regimens given in a trial. We start by recalling the selection criterion proposed by Mozgunov and Jaki (2019) in case of binary toxicity and binary efficacy endpoints.

### 2.1 Selection criterion

Following Mozgunov and Jaki (2019), let us consider a random variable *Y* that takes one of three values corresponding to (i) “efficacy and no toxicity,” (ii) “no efficacy and no toxicity” and (iii) “toxicity.” Outcomes “toxicity and no efficacy” and “toxicity and efficacy” are combined as it is assumed that efficacy can only be observed when no toxicity occurs. Let $Z=\left[Z^{\left(1\right)},Z^{\left(2\right)},Z^{\left(3\right)}\right]\in S^{2}$ be a random vector defined on the triangle

$$S^{2}=\left\{Z:Z^{\left(1\right)}>0,\;Z^{\left(2\right)}>0,Z^{\left(3\right)}>0;\sum_{i=1}^{3}Z^{\left(i\right)}=1\right\},$$

where $Z^{\left(1\right)},Z^{\left(2\right)},Z^{\left(3\right)}$ correspond to probabilities of each of the three outcomes. Assume that **Z** has a prior Dirichlet distribution Dir(**v**), $v={\left[v_{1},v_{2},v_{3}\right]}^{T}\in R_{+}^{3}$ where $v_{i}>0$. After *n* realizations in which $x_{i}$ outcomes of *i*, i=1,2,3 are observed, the posterior probability density function, $f_{n}$, of a random vector $Z^{\left(n\right)}$ follows a Dirichlet distribution again,  Dir (v+x), where $x=[x_{1},x_{2},x_{3}]$, $\sum_{i=1}^{3}x_{i}=n$. Denote the vector in the neighbourhood of which the probability density function $f_{n}$ concentrates as n→∞ by $\theta ={\left[\theta_{1},\theta_{2},\theta_{3}\right]}^{T}$, where θ_1_ is the probability of “efficacy and no toxicity,” θ_2_ is the probability of “no efficacy and no toxicity” $\theta_{3}=1-\theta_{1}-\theta_{2}$ is the probability of toxicity.

Let the target be the regimen with probabilities of the three outcomes equal to $\gamma =\left[\gamma_{1},\gamma_{2},\gamma_{3}\right]\in S^{2}$ where $\gamma_{1},\gamma_{2},\gamma_{3}$ are the target probabilities of “no toxicity and efficacy,” “no toxicity and no efficacy” and “toxicity,” respectively. Then, the goal is to find the regimen which characteristics (probabilities of these events) as close as possible to the targets. Mozgunov and Jaki (2019) proposed to use the weighted Shannon entropy to compute the statistical gain in such experiment. It was shown by Mozgunov and Jaki (2018b) that the statistical gain is nearly maximised when

(1) $$\delta \left(\theta ,\gamma \right):=\frac{\gamma_{1}^{2}}{\theta_{1}}+\frac{\gamma_{2}^{2}}{\theta_{2}}+\frac{{\left(1-\gamma_{1}-\gamma_{2}\right)}^{2}}{1-\theta_{1}-\theta_{2}}-1$$

is minimised. The measure given in Equation (1) consists of contributions from three terms that correspond to three possible outcomes considered under the model and have the following interpretations:

- When θ_1_ tends to 0 the regimen is either inefficacious or (and) highly toxic. Then, the value of the function tends to infinity meaning that the treatment should be avoided.
- When θ_2_ tends 0 the regimen is either highly efficacious or (and) highly toxic. Then, this term penalises the high toxicity regardless of high efficacy as the function tends to infinity. This term prevents the selection of highly toxic regimens.
- When $1-\theta_{1}-\theta_{2}$ tends 0, the regimen is associated with nearly no toxicity. However, the optimal regimen is expected to be associated with nonzero toxicity. Then, this terms drives the allocation away from the underdosing regimens.

Note that all terms are dependent — an increase in one leads to decreases in others. The optimal value is attained at the point of target characteristics only. This measure was proposed as a trade‐off function to govern the regimen selection in a Phase I/II clinical trial.

The trade‐off function (1) depends on probabilities θ_1_ and θ_2_ while the goal of a Phase I/II clinical trial is conventionally formulated in terms of toxicity ($p_{t}$) and efficacy ($p_{e}$) probabilities and corresponding targets $\gamma_{t}$ and $\gamma_{e}$. Employing the assumption of independence of toxicity and efficacy, we re‐parametrise (1) using $\theta_{1}=\left(1-p_{t}\right)p_{e}$, $\theta_{2}=\left(1-p_{t}\right)\left(1-p_{e}\right)$, $\gamma_{1}=\left(1-\gamma_{t}\right)\gamma_{e}$ and $\gamma_{2}=\left(1-\gamma_{t}\right)\left(1-\gamma_{e}\right)$ and denote the trade‐off function by $\delta (p_{t},p_{e},\gamma_{t},\gamma_{e})$. The trade‐off function can be computed for each of the *m* regimens under study with parameters $p_{t,1},\ldots ,p_{t,m}$ and $p_{e,1},\ldots ,p_{e,m}$, respectively. Given $\delta (p_{t,j},p_{e,j},\gamma_{t},\gamma_{e})$, j=1,…,m, the target regimen $j^{*}$ satisfies

(2) $$\delta \left(p_{t,j^{*}},p_{e,j^{*}},\gamma_{t},\gamma_{e}\right)=\min_{j=1,\ldots ,m}\delta \left(p_{t,j},p_{e,j},\gamma_{t},\gamma_{e}\right).$$

In the function (1), the probability of efficacy $p_{e}$ belongs to the unit interval (0,1). In order to apply the function (1) to govern the regimen selection in trials with a continuous efficacy endpoint, the core idea of this work is to benchmark a continuous endpoint by transforming it into a probability scale via the logistic transformation. The question of transformation is considered in the following section.

### 2.2 Transformation for continuous endpoint

In Statistics, a conventional choice of transformation of a continuous variable ξ defined on the whole real line R to the unit interval (0,1) is the logistic transformation (also known as inverse logit)

(3) $$T\left(\xi \right)\equiv {\mathrm{logit}}^{-1}\left(\xi \right)=\frac{\exp\left(\alpha +\beta \xi \right)}{1+\exp\left(\alpha +\beta \xi \right)}$$

for given values of α and β (Hirakawa, 2012; Mozgunov, Jaki & Gasparini, 2019), where

$$\mathrm{logit}\left(p_{e}\right)=\log\frac{p_{e}}{1-p_{e}}.$$

We propose to use the logistic transformation (3) to map the variable ξ∈R to $p_{e}\in \left(0,1\right)$.

Note that a similar problem of mapping variables is common in drug‐benefit analysis. There are several factors of benefits a drug can provide which, however, can be defined on different scales. To ensure that one indicator of benefit does not prevail due to the fact that it is defined for larger values, a linear transformation $\frac{\xi -\psi }{\psi^{\prime }-\psi }$ is conventionally used, where ξ is defined on an interval $\left(\psi ,\psi^{\prime }\right)$ and ψ is the lowest efficacy bound and $\psi^{\prime }$ is the highest possible value of ξ. This, however, involves a truncation of a random variable that can be avoided by using the logistic transformation.

The crucial part of the transformation (3) are parameters α and β which should be chosen given the clinical context of the efficacy outcome. The parameter α defines the shift of the transformation. This largely depends on “where sensible values of ξ start” or, in other words, on the lowest efficacy bound ψ. The parameter β corresponds to the slope of the transformation and depends on the width of a possible range of ξ. Values β<0 means that lower values of ξ correspond to better characteristics of a drug, and β>0 otherwise. We propose the following guideline for the choice of the parameters.

As ψ is the lowest efficacy bound, it is natural to set the value of the corresponding transformation to some small value on (0,1) scale, say, 0.01. Similarly, let $\psi^{\prime }$ be the highest clinically possible value for efficacy outcome. Then, we set the value of the corresponding transformation to a value close to 1, for example, 0.9. This results in two conditions for the transformation

$$\left\{\begin{array}{c} T(\psi )=0.01\\ T(\psi^{\prime })=0.90, \end{array}\right.$$

which are solved for

(4) α∗= logit 0.01−β∗ψβ∗= logit 0.9− logit 0.01ψ′−ψ.

Note that values 0.01 and 0.9 above are, to some extent, arbitrary. The value 0.01 is chosen as it is common to specify a lower bound ψ in Phase I/II clinical trials, while the value 0.9 allows for values of the original efficacy endpoint ξ to be higher than specified $\psi^{\prime }$. In contrast to the linear transformation, the proposed logistic function allows for values ξ<ψ and $\xi >\psi^{\prime }$ avoiding the truncation.

While the logistic transformation allows not to truncate the variable of interest ξ (compared to the linear transformation), its specific choice in this communication is due to the common application of it in Statistics. There are other alternative transformations mapping the outcome of interest to the unit interval. Specifically, one can use the cumulative distribution function (CDF) of the standard normal distribution (Φ‐link)

$$T^{\prime }\left(\xi \right)=\Phi \left(\alpha +\beta \xi \right)$$

or the inverse complementary log‐log transformation

$$T^{\prime \prime }\left(\xi \right)=1-\exp\left[-\exp\left(\alpha +\beta \xi \right)\right].$$

The main difference is how the chosen transformation approaches the boundaries of the interval. For instance, as the outcome of interest tends to its the most desirable value, the inverse complementary log‐log transformation approaches 1 faster than either the logistic transformation or Φ‐link. Comparing the latter two, the logistic transformation has slightly flatter tails, i.e the respective curve (with same parameters α and β) approaches the axes slower. In the simulations below, we will consider the logistic transformation only but we provide the results for two other transformations in Supporting Information using the same set of parameters. It is found that all of these transformations lead to similar operating characteristics in all considered scenarios, and the final choice is up to the statistician and clinician which should follow the most plausible interpretation. Once the transformation is defined, we propose the following design for Phase I/II clinical trials with binary toxicity and continuous efficacy endpoints.

## 3 DESIGN

### 3.1 Regimen‐finding design

Let *m* be a total number of regimens in Phase I/II trial and *N* is a total number of patients. The goal of the trial is to find the OBR corresponding to a toxicity probability below the upper toxicity bound ϕ and to the highest efficacy above the lowest efficacy bound ψ. Motivated by relaxing any parametric and monotonicity assumptions between regimens, we consider each regimen independently and do not allow borrowing as the model assumes that the probabilities of toxicity (and efficacy outcomes) are drawn from different distributions, and we have a reason to think that regimens are systematically different. We employ this assumption to apply the design in various clinical trial settings where the ordering of toxicity/efficacy might not be known, and the parameters corresponding to various regimens might be noticeably different.

Let $B(\nu_{j},\beta_{j}-\nu_{j})$ be a Beta prior distribution of the probability of toxicity $p_{t,j}$ corresponding to regimen *j* where $\beta_{j},\nu_{j}>0$. Let ξ be a continuous efficacy endpoint having a normal distribution $N(\mu_{j},\sigma_{j}^{2})$ where $\left(\mu_{j},\sigma_{j}^{2}\right)$ have an Normal‐inverse Gamma Distribution with parameters ${\mathrm{NG}}^{-1}\left(\mu_{0,j},\lambda_{j},\zeta_{j},\zeta_{j}^{\prime }\right)$. Here the parameters have the following interpretation: $\mu_{0,j}$, $\zeta_{j}^{\prime }$ are prior mean and prior sum of squared deviations, $\lambda_{j}$ and $\zeta_{j}$ are “strength” of the mean and squared deviation, respectively, in terms of number of observations. Assume that $n_{j}$ patients have been assigned to regimen *j* and $t_{j}$ toxicity outcomes and corresponding efficacy outcomes $(x_{j}^{\left(1\right)},\ldots ,x_{j}^{\left(n_{j}\right)}$) have been observed. Then, the probability of toxicity has a posterior Beta distribution $B(\nu_{j}+t_{j},\beta_{j}+n_{j})$ with mean

$${\hat{p}}_{t,j}=\frac{t_{j}+\nu_{j}}{n_{j}+\beta_{j}}$$

and the parameters of the efficacy outcomes $\left(\mu_{j},\sigma_{j}^{2}\right)$ have a posterior Normal‐inverse gamma distribution with parameters

$$\left(\hat{\mu_{j}}=\frac{\lambda_{j}\mu_{0,j}+{\bar{x}}_{j}}{\lambda_{j}+n_{j}},\;\lambda_{j}+n_{j},\;\zeta_{j}+\frac{n_{j}}{2},\;\zeta^{\prime }+\frac{1}{2}\sum_{i=1}^{n_{j}}\left(x_{j}^{\left(i\right)}-{\bar{x}}_{j}\right)+\frac{n_{j}\lambda_{j}}{\lambda_{j}+n_{j}}\frac{{\left({\bar{x}}_{j}-\mu_{0}\right)}^{2}}{2}\right)\;,$$

where ${\bar{x}}_{j}=\sum_{i=1}^{n_{j}}x_{j}^{\left(i\right)}$ is a sample mean and ${\hat{\mu }}_{j}$ is the posterior mean. Then, by plugging‐in the posterior mean of the toxicity probability ${\hat{p}}_{t,j}$ distribution and the transformation of the posterior mean of the efficacy outcome distribution, $T(\hat{\mu_{j}})$, for some fixed values of α and β in Equation (3) one can obtain the “plug‐in” estimator of the trade‐off function (1) for regimen *j*

(5) $${\hat{\delta }}_{j}^{\left(n_{j}\right)}=\frac{{\left(\gamma_{e}\left(1-\gamma_{t}\right)\right)}^{2}}{T\left(\hat{\mu_{j}}\right)\left(1-{\hat{p}}_{t,j}\right)}+\frac{{\left(\left(1-\gamma_{e}\right)\left(1-\gamma_{t}\right)\right)}^{2}}{\left(1-T\left(\hat{\mu_{j}}\right)\right)\left(1-{\hat{p}}_{t,j}\right)}+\frac{\gamma_{t}^{2}}{{\hat{p}}_{t,j}}-1,$$

where $\gamma_{t}$ and $\gamma_{e}$ are the target levels of toxicity and efficacy, respectively chosen by clinicians. The trade‐off function is estimated for all regimens in the study, j=1,…,m. Then, the design proceeds as follows.

Patients are assigned sequentially cohort‐by‐cohort, where a cohort is a small group of typically 1 to 4 patients. After the outcomes for the last cohort are obtained and $n_{1},\ldots ,n_{m}$ patients were assigned to regimens 1,…,m, respectively, the estimates of the trade‐off function ${\hat{\delta }}_{1}^{\left(n_{1}\right)},\ldots ,{\hat{\delta }}_{m}^{n_{m}}$ are obtained as in Equation (5). To allow for a better exploration of the regimen–efficacy and regimen–toxicity relationships (Mozgunov & Jaki, 2019; Thall & Wathen, 2007; Wages & Tait, 2015) the next cohort of patients is randomised between two “best” regimens corresponding to the minimum and second minimum of ${\hat{\delta }}_{1}^{\left(n_{1}\right)},\ldots ,{\hat{\delta }}_{m}^{n_{m}}$. The randomisation probabilities are proportional to $1/{\hat{\delta }}_{j}^{\left(n_{j}\right)}$. The design proceeds until the maximum number of patients, *N*, have been treated. The regimen $j^{*}$ satisfying

(6) $${\hat{\delta }}_{j^{*}}^{\left(n_{j^{*}}\right)}=\min_{j=1,\ldots ,m}{\hat{\delta }}_{j}^{\left(n_{j}\right)}$$

is adopted as the final recommendation.

Importantly, this work focuses on the setting where the target of the trial is to find the OBR defined as the regimen corresponding to the highest efficacy amongst safe regimens. The selection criterion (6), however, aims to find the regimen whose characteristics are as close as possible to $\gamma_{t},\gamma_{e}$ and hence specific target values are required. We therefore use the best possible targets (highly efficacious and very low toxicity risk, i.e., $\gamma_{e}=0.99$ and $\gamma_{t}=0.01$). This choice means that we would like to have as high efficacy as possible with very low toxicity. This part drives the allocation of patients. In addition, safety and futility constraints are used to ensure the correct regimen is targeted.

### 3.2 Safety and futility constraints

For ethical reasons, it is required to control the number of patients exposed to such regimens and two time‐varying constraints similar to proposed by Mozgunov and Jaki (2019) are introduced.

A regimen *j* is considered to be unsafe if after $n_{j}$ patients

(7) $$\int_{\phi }^{1}f_{t,j}^{\left(n_{j}\right)}\left(p\right)dp\geq \eta_{1}^{\left(n_{j}\right)},$$

where $f_{t,j}^{\left(n_{j}\right)}$ is the Beta posterior density function of the toxicity probability, ϕ is the upper toxicity bound and $\eta_{1}^{\left(n_{j}\right)}$ is the probability that controls overdosing. For the constraint to become more strict as the trial progresses, a nonincreasing function of $n_{j}$ for $\eta_{1}^{\left(n_{j}\right)}$ is used. Subsequently, we use a linear decreasing function $\eta_{1}^{\left(n_{j}\right)}=\max\left(0.95-r_{t}n_{j},{\bar{\eta }}_{1}\right)$ where 0.95 is a controlling probability before any data is available, $r_{t}>0$ is the rate of decreasing and ${\bar{\eta }}_{1}$ is the controlling probability for the final recommendation in the trial. These safety constraint parameters $r_{t}$ and ${\bar{\eta }}_{1}$ can either be specified by experts or alternatively calibrated with respect to a trial's goals using simulations.

Similarly, we use the following time‐varying futility constraint. A regimen *j* is considered to be futile if after $n_{j}$ patients

(8) $$\int_{-\infty }^{\psi^{*}}f_{e,j}^{\left(n_{j}\right)}\left(x\right)dx\geq \eta_{2}^{\left(n_{j}\right)},$$

where $f_{e,j}^{\left(n_{j}\right)}$ is a density of a Normal distribution with mean ${\hat{\mu }}_{j}$ and variance

$$\left(\zeta^{\prime }+\frac{1}{2}\sum_{i=1}^{n_{j}}\left(x_{j}^{\left(i\right)}-{\bar{x}}_{j}\right)+\frac{n_{j}\lambda_{j}}{\lambda_{j}+n_{j}}\frac{{\left({\bar{x}}_{j}-\mu_{0}\right)}^{2}}{2}\right)/\left(\left[\zeta_{j}+\frac{n_{j}}{2}\right]\left[\lambda_{j}+n_{j}\right]\right),$$

which is the variance of the posterior Normal‐inverse gamma distribution as above, $\psi^{*}$ is the efficacy threshold and $\eta_{2}^{\left(n_{j}\right)}$ is the controlling probability which is an increasing function of $n_{j}$. We use a linear increasing function $\eta_{2}^{\left(n_{j}\right)}=\max\left(0.8-r_{e}n_{j},{\bar{\eta }}_{2}\right)$ where 0.8 is a controlling probability before any data is available, $r_{e}>0$ is the rate of decreasing and ${\bar{\eta }}_{2}$ is the controlling probability for the final recommendation in the trial. Note that the futility constraint (8) assumes that higher values of the outcome correspond to a better performance of the drug. In other cases, the bounds of the integration should be changed to $\psi^{*}$ and +∞, respectively. The normal approximation in the constraint (8) is adapted due to its simplicity and inclusion of many statistical software.

Finally, we require the design escalation/de‐escalation decisions to satisfy the coherence principles formulated by Cheung (2005) with respect to the known ordering of toxicities (Mozgunov & Jaki, 2019).

## 4 NUMERICAL STUDY

### 4.1 Setting

In this section, we investigate the performance of the proposed design and compare it to the performance of the design by Hirakawa (2012) in the setting of the squamous cell carcinoma Phase I trial. We consider two cases investigated by Hirakawa (2012): single‐agent and dual‐agent combination trials. There are four dose levels of drug *A* ($d_{A}^{1},d_{A}^{2},d_{A}^{3},d_{A}^{4}$) which are given as monotherapies in the single‐agent setting and in combinations with two dose levels of drug *B* ($d_{B}^{1},d_{B}^{2})$. This results in four and eight regimens considered in each case, respectively. The simulation scenarios were constructed based on real experimental squamous cell carcinoma antigen (SCCA) data (see Hirakawa, 2012, for details of the original clinical trial).The efficacy outcomes is a change in log‐transformed SCCA levels at baseline and by the end of the treatment. Consequently, the lower values of the efficacy outcomes correspond to a better performance of the agents. The upper toxicity is ϕ=0.3 and the upper efficacy bound is ψ=0 corresponding to no changes in SCCA levels. The number of patients in the single agent trial is N=36 and N=72 in the combination one, and the trials are required to start at the lowest dose (combination). The cohort size is fixed to be 3. The goals are to find the OBR.

We choose the same set of scenarios (Table 1) as in the original work. The efficacy outcomes have a Normal distribution $N(\mu_{j},1)$ where $\mu_{j}$ are specified by a scenario. The toxicity of both monotherapy and combination increases with the dose of each agent, but the efficacy can be either strongly increasing (scenarios 1–3 and 7–8) or can have a plateau (scenarios 4 and 9). Note that under scenarios 1–3, toxicity and efficacy monotonically increase and hence these scenarios can be used to assess the potential loss in identifying the OBR compared to designs employing parametric models for regimen–toxicity and regimen–efficacy relationships. Scenarios 5 and 6 are considered to investigate the ability of the designs to terminate the trial when there is no efficacious or no safe dose, respectively. As originally considered, we assume a weak correlation r=0.2 between toxicity and efficacy outcomes, no delayed responses and that efficacy can be observed regardless the toxicity. We investigate the influence of these assumptions in Section 5.

**Table 1:** True values of $\left(p_{t,j},\mu_{j}\right)$ for each dose levels of a single agent $d_{A}$ (scenarios 1–6) and for each combination of two agents, $d_{A}$ and $d_{B}$ (scenarios 7–9)

		$d_{A}^{1}$	$d_{A}^{2}$	$d_{A}^{3}$	$d_{A}^{4}$
Scenario 1		(0.01, 0.5)	(0.15,−0.5)	(0.45, −1.5)	(0.65, −3.0)
Scenario 2		(0.05,−0.5)	(0.50, −0.6)	(0.60, −0.7)	(0.70, −0.8)
Scenario 3		(0.01, 0.5)	(0.03, −0.5)	(0.05, −1.5)	(0.08,−3.0)
Scenario 4		(0.01, 0.5)	(0.10,−2.0)	(0.30, −2.0)	(0.60, −2.0)
Scenario 5		(0.01, 2.0)	(0.05, 2.0)	(0.10, 2.0)	(0.15, 2.0)
Scenario 6		(0.50, 0.0)	(0.60, −0.3)	(0.70, −0.7)	(0.80, −1.0)
Scenario 7	$d_{B}^{1}$	(0.01, 0.5)	(0.10, 0.0)	(0.40, −1.5)	(0.50, −2.5)
	$d_{B}^{2}$	(0.05, −1.5)	(0.15,−2.0)	(0.45, −3.5)	(0.55, −4.5)
Scenario 8	$d_{B}^{1}$	(0.01, 0.0)	(0.05, −0.5)	(0.15,−3.5)	(0.45, −5.5)
	$d_{B}^{2}$	(0.45, −1.0)	(0.50, −1.5)	(0.60, −4.5)	(0.90, −6.5)
Scenario 9	$d_{B}^{1}$	(0.01, 0.0)	(0.15,−2.0)	(0.40, −2.0)	(0.50, −2.0)
	$d_{B}^{2}$	(0.05, 0.0)	(0.20, −2.0)	(0.45, −2.0)	(0.55, −2.0)

The OBR is in bold.

In the analysis, we focus on (i) the proportion of optimal regimen selection, (ii) proportion of toxic responses and (iii) mean efficacy response. The study is performed using R (R Core Team, 2015) and 10,000 replications for each scenario. The design by Hirakawa (2012) is specified as in the original publications and referred as “Emax” as the Emax model is used for the efficacy relationship. For completeness, we also include the design by Yeung et al. (2015) into the comparison in the setting of monotherapy (refereed as “Gain Design (GD).” The parameters of this design are set as in the original work. We compare the characteristics in the combination setting to the design by Hirakawa (2012). To assess the performance comprehensively and to take into account the “difficulty” of the scenarios, the benchmark for binary toxicity and continuous efficacy by Mozgunov, Jaki and Paoletti (2018b) is also included. The vectors of complete information are generated as proposed in the original work. The estimates are then plugged‐in into the trade‐off function (5) used by the WE design. Source R code implementing the proposed design is available on GitHub (https://github.com/dose‐finding/cont‐phase‐I‐II) and as Supporting Information.

### 4.2 Design specification

The proposed design is formulated in terms of regimens and does not require any model. Therefore, it can be straightforwardly applied to both single agent and combination trials with minor technical differences. We specify the parameters of the proposed design below.

**Figure 1:**
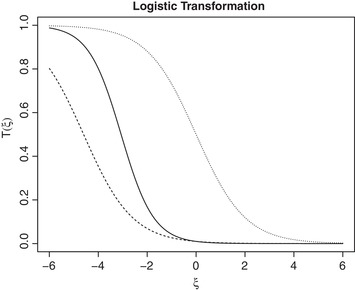
The logistic transformation (3) for (i) $\alpha^{*}=0,\beta^{*}=-1$ (dotted line), (ii) $\alpha^{*}\;=\;-4.6,\beta^{*}=-1$ (dashed line) and (iii) $\alpha^{*}=-4.6,\beta^{*}=-1.5$ (solid line)

First, the parameters of the logistic transformation (3) are to be defined. Following the clinical context, the largest clinically significant value (corresponding to a poor performance) is ψ=0 and the lowest value that can be observed for the treatment (based on the actual clinical trials) is $\psi^{\prime }=-4.5$ (Hirakawa, 2012). Using (4), the values of parameters to be used in the transformation are α≈−4.6 and β≈−1.5. The graph of the proposed logistic transformation is given in Figure 1.

The dotted line in Figure 1 corresponds to the logistic transformation with conventional parameters $\alpha^{*}=0,\beta^{*}=-1$ — the curve is decreasing as higher values of response correspond to a worse performance. First of all, using the information that the clinically relevant values start at ξ=0, one can find a clinically meaningful value of $\alpha^{*}$ correspond to the shift. The dashed line corresponding to $\alpha^{*}=-4.6,\beta^{*}=-1$ satisfies this clinical interpretation. Furthermore, based on preclinical information the response higher than −4.5 is not expected. Therefore, by adjusting $\beta^{*}$ one can achieve a better distinction between different efficacy levels through the higher value of $\beta^{*}=-1.5$ (solid line).

Second, the parameters of Beta prior and Normal‐inverse gamma distribution to be used for each regimen. We set the “strength” of each prior for all regimens to be equal to $\beta_{1}=\cdots =\beta_{4}=1$ and $\lambda_{1}=\cdots =\lambda_{4}=1$. Then, the selection at the beginning of the trial are defined by prior parameters $\nu_{1},\ldots ,\nu_{4}$ (for toxicity) and $\mu_{0,1},\ldots ,\mu_{0,4}$ (for efficacy) which are equal to the mean prior toxicity and mean prior efficacy for the specified levels of β and λ. Note that the choice of these values correspond to the problem of a “skeleton” choice in the setting of the Continual Reassessment Method (O'Quigley, Pepe & Fisher, 1990, CRM). Specifically, it is argued by O'Quigley and Zohar (2010) that the CRM can identify the target dose if the spacings between skeleton values are “adequately chosen.” Essentially, the same holds for the proposed designs (Mozgunov & Jaki, 2019), and the “adequate” choice of the prior toxicity and mean prior efficacy are calibrated as follows.

We assume a linear increase (decrease) in the prior toxicity (efficacy) estimates. Formally, we investigate the following prior parameters $p_{1},\ldots ,p_{4}$ (for toxicity) and $\mu_{0,1},\ldots ,\mu_{0,4}$ (for efficacy):

pj=start.tox+(j−1)×step.tox, and μj=start.eff+(j−1)×step.eff

where start.tox,start.eff,step.tox,step.eff are parameters that will be varied to define different prior choices. We use a grid search over various combinations of these four parameters. Specifically, we consider the following values

start.tox={0.05,0.10,0.15},start.eff={−0.80,−0.90,…,−1.20}

step.tox={0.03,0.04,…,0.10},step.eff={−0.01,−0.025,−0.05,−0.075,−0.1}.

This results in 600 sets of prior parameters to be considered. We restrict the search to the set of parameters that preserve the initial ordering (1, 2, 3, 4) assumed to be specified by a clinical prior to trial. This ordering can be interpreted as an initial ordering that a clinician would like to preserve in the trial which, however, can change as the trial progresses.

We use two scenarios for the calibration: scenario 2 and scenario 3 as corresponding to different clinical scenarios with the first and the last regimens being the OBR, respectively. Then, for each choice of the set of parameters and under each scenario, we run 10,000 simulations of the design (with no safety or futility constraints). We are interested in the proportion of the OBR selections. To find the set of parameters that leads to an accurate selection in both scenarios, we use the geometric mean of the proportion of correct selections as the criterion. The maximum value of the geometric mean corresponds to the choice of parameters

(9) $${\hat{p}}_{t}=\left(0.10,0.14,0.18,0.22\right),\;\;\;\mu_{0}=\left(-1.00,-1.025,-1.05,-1.075\right).$$

**Figure 2:**
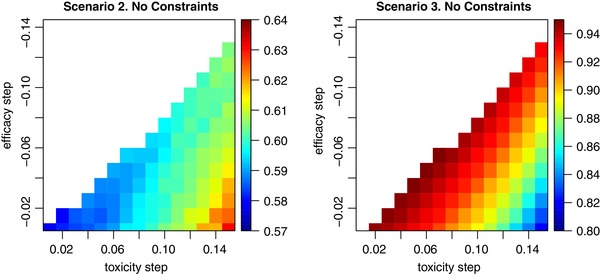
Proportion of OBR selections under different combinations of toxicity and efficacy steps

However, there were 136 more sets of parameters that resulted in the geometric mean within 1.5% of the maximum value. Consequently, there are many possible choices of the prior parameters that lead to the accurate OBR selection under these scenarios. To demonstrate the sensitivity of the performance to the choice of the step between toxicity and efficacy prior estimates, we fix the starting values of toxicity and efficacy as above, and vary the steps between regimens only step.tox={0.01,0.02,…,0.15},step.eff={−0.005,−0.015,…,−0.145}. The proportions of OBR selections using each combination of toxicity and efficacy steps under two scenarios are given in Figure 2. The white colour in Figure 2 corresponds to the sets of prior parameters that do not preserve the specified order, and therefore, are excluded from the consideration.

Under the specified initial ordering, the design identifies the OBR with higher probability under scenario 2 if the step between prior toxicity means is larger (for the fixed step in prior efficacy estimates). Indeed, given that the OBR is regimen 1 and it is the starting regimen according to the specified order, a larger step in toxicity makes the probability of escalating (with respect to the specified order) smaller. Note, however, that the performance varies between 57% and 64%. At the same time, for the fixed efficacy step, a higher toxicity leads to a lower proportion of the OBR selections under scenario 3 with the OBR being the regimen 4. Again, under the specified order, a larger step makes it more difficult for the design to reach regimen 4. Given the opposite effect under these scenarios, the calibration allows for finding a better trade‐off. We refer the reader to Supporting Information for the details of the sensitivity analysis of the design to the choice of prior parameters when the safety and efficacy constraints are imposed.

Consequently, the prior means of toxicity and efficacy as specified in Equation (9) were used in the single‐agent setting, and the prior parameters

$${\hat{p}}_{t}=\left[\begin{array}{cccc} 0.10 & 0.14 & 0.18 & 0.22\\ 0.14 & 0.18 & 0.22 & 0.26 \end{array}\right],$$

$$\mu_{0}=\left[\begin{array}{cccc} -1.000 & -1.025 & -1.05 & -1.075\\ -1.025 & -1.05 & -1.075 & -1.100 \end{array}\right]$$

were used in the combination trial. The parameters $\zeta_{j},\zeta_{j}^{\prime }$ affect the futility constraint only and were found not to affect the design in a reasonable range of values. We consequently fix $\zeta_{1}=\cdots =\zeta_{4}=2$ and $\zeta_{1}^{\prime }=\cdots =\zeta_{4}^{\prime }=3$.

Finally, the parameters of the safety and futility constraints (7)–(8) are to be specified. The parameters of the safety and futility constraints were calibrated over the set if single‐agent scenarios and the following values were subsequently used: $r_{t}=r_{e}=0.02,{\bar{\eta }}_{1}^{\left(n_{j}\right)}=0.6,{\bar{\eta }}_{2}^{\left(n_{j}\right)}=0.3,\psi^{*}=0.20$. One can check that under all considered scenarios, the minimum of the escalation criterion (5) (i.e., the most desirable regimen) is indeed the true OBR as defined by the trial. We will refer to the proposed design as “WE.” The performance of the design is studied in the following section.

### 4.3 Operating characteristics

The proportions of each regimen selections, proportion of toxicity responses, and mean toxicity responses by the proposed WE design, the Emax design and GD in the single‐agent scenarios are given in Table 2. Results for Emax and GD approaches are obtained from the original works.

**Table 2:** Operating characteristics of the proposed WE design, the Emax design, and the Gain Design (GD): proportion of each regimen under scenarios 1–6

Design		$d_{A}^{1}$	$d_{A}^{2}$	$d_{A}^{3}$	$d_{A}^{4}$	Termination	Toxicity	Efficacy
Scenario 1								
Benchmark		0.1	**95.6**	2.9	0.0	1.5		
WE		1.5	**82.0**	6.2	0.1	10.3	20.9	−0.6
Emax		15.7	**80.1**	2.8	0.0	1.4	15.2	−0.4
GD		0.0	**59.9**	39.5	0.6	0.0	18.0	−0.5
Scenario 2								
Benchmark		**99.8**	0.0	0.0	0.0	0.2		
WE		**96.9**	1.9	0.1	0.0	1.1	20.0	−0.5
Emax		**96.8**	1.4	0.0	0.0	1.8	10.5	−0.5
GD		**94.3**	0.7	0.0	0.0	5.0	5.4	−0.5
Scenario 3								
Benchmark		0.0	0.0	0.0	**100.0**	0.0		
WE		0.0	1.0	7.6	**91.3**	0.2	6.3	−2.2
Emax		2.4	2.8	4.1	**90.7**	0.0	5.3	−2.2
GD		1.0	0.5	0.6	**97.9**	0.0	5.4	−1.7
Scenario 4								
Benchmark		0.0	**99.9**	0.0	0.0	0.1		
WE		0.0	**81.7**	15.6	0.0	2.6	15.8	−1.7
Emax		4.8	**73.5**	20.0	0.0	1.7	14.8	−1.7
GD		0.1	**34.4**	62.2	3.3	0.0	17.1	−1.5
Scenario 5								
Benchmark		0.0	0.0	0.0	0.0	**100.0**		
WE		0.0	0.0	0.0	0.0	**100.0**	8.8	2.0
Emax		0.4	0.0	0.0	0.0	**99.6**	2.6	2.0
GD		13.2	0.3	5.3	81.1	**0.01**	9.3	2.0
Scenario 6								
Benchmark		0.2	0.0	0.0	0.0	**99.8**		
WE		0.5	0.0	0.0	0.0	**99.5**	52.2	−0.1
Emax		0.1	0.0	0.0	0.0	**99.9**	52.6	−0.1
GD		1.7	0.0	0.0	0.2	**98.1**	53.0	−0.1

The columns “Termination,” “Toxicity” and “Efficacy” correspond to the proportion of earlier termination by each design, the proportion of average toxicity response and the average efficacy response, respectively. Proportion of the OBR are in bold. Results are based on 10^4^ replicated trials for WE and the benchmark and on 2,000 for Emax and GD.

Regarding the proportion of correct selections in single‐agent scenarios, the WE design performs comparably to the Emax design under all scenarios with strictly increasing dose–efficacy relationship (scenarios 1–3) and find the OBR with probability over 80%. Moreover, the ratios of the proportion of correct selections with respect to the optimal benchmark is always above 85%. The proposed methods, however, offers a substantial gain in scenario 4 with a plateau in a dose‐toxicity curve —it outperforms Emax by nearly 10% while performing comparable in terms of the mean number of toxicities and mean number of efficacies. WE favours a dose at the beginning of the plateau and avoids exposing patients to a dose with the same efficacy, but higher toxicity. Importantly, it also does not recommend the inefficacious dose $d_{A}^{1}$ compared to 5% proportion of selections by Emax. Compared to the GD, the WE design performs comparably under under scenario 2 while outperforming by 22% and 46% under scenarios 1 and 4, respectively. At the same time, GD leads to a more accurate OBR selection under scenario 3 (97.9% against 91.3% by the WE design) but the margin is lower than the benefit the WE design can offer under other scenarios. While the WE design shows a noticeable improved performance, it is not relatively close to the benchmark's performance which finds the OBR nearly in all simulated trials. This might be a consequence of not modelling the plateau in the dose–efficacy relationship which the benchmark can identify by the construction. Nevertheless, the ratio of correct selections for WE is still high, nearly 82%.

Regarding the proportion of toxicity outcomes, it is controlled below the maximum acceptable level for all designs under all scenario. The major difference in the proportion of toxic responses compared to the Emax and GD can be found under scenario 2 with a large jump in toxicity probabilities after dose $d_{A}^{1}$. Due to the explicit regimen–efficacy model, Emax and GD identify the jump in toxicities faster which results in fewer patients assigned to higher doses than the WE design. At the same time, the average toxicity level is still controlled (20%) and is far below the acceptable threshold 30%. Finally, both Emax and WE designs perform comparably under scenarios 5–6 with no OBR and able to terminate the trial earlier in nearly 100% of trials, while the GD almost never terminates under scenario 5. As pointed out by Yeung et al. (2015), this is because no minimum efficacy requirement is included in the GD approach.

The operating characteristics of both designs in the combination scenarios are given in Table 3.

**Table 3:** Operating characteristics of the proposed WE design and the Emax design: proportion of each regimen selections under scenarios 7–9

Design		$d_{A}^{1}$	$d_{A}^{2}$	$d_{A}^{3}$	$d_{A}^{4}$	Termination	Toxicity	Efficacy
Scenario 7								
Benchmark	$d_{B}^{1}$	0.0	0.0	0.0	0.0			
	$d_{B}^{2}$	0.0	**99.9**	0.1	0.0			
WE	$d_{B}^{1}$	0.0	0.1	0.7	0.3	0.4	14.1	−1.6
	$d_{B}^{2}$	24.8	**73.1**	0.6	0.0			
Emax	$d_{B}^{1}$	0.1	5.1	3.4	0.0	1.8	14.9	−1.6
	$d_{B}^{2}$	14.8	**70.1**	4.7	0.0			
Scenario 8								
Benchmark	$d_{B}^{1}$	0.0	0.0	**99.9**	0.1			
	$d_{B}^{2}$	0.0	0.0	0.0	0.0			
WE	$d_{B}^{1}$	1.0	12.0	**85.2**	0.7	0.5	17.1	−2.8
	$d_{B}^{2}$	0.5	0.0	0.0	0.0			
Emax	$d_{B}^{1}$	4.3	11.5	**78.6**	3.1	2.1	17.3	−2.3
	$d_{B}^{2}$	0.0	0.1	0.3	0.0			
Scenario 9								
Benchmark	$d_{B}^{1}$	0.0	**99.9**	0.1	0.0			
	$d_{B}^{2}$	0.0	0.0	0.0	0.0			
WE	$d_{B}^{1}$	2.8	**62.1**	2.0	0.0	2.5	16.8	−1.7
	$d_{B}^{2}$	3.7	26.9	0.0	0.0			
Emax	$d_{B}^{1}$	0.9	**44.9**	2.8	0.0	0.0	16.7	−1.5
	$d_{B}^{2}$	4.3	45.3	1.8	0.0			

The columns “Termination,” “Toxicity” and “Efficacy” correspond to the proportion of earlier termination by each design, the proportion of average toxicity response and the average efficacy response, respectively. Proportion of the OBR selections are in bold. Results are based on 10^4^ replicated trials for WE and the benchmark and on 2,000 for Emax.

In combination scenarios, the novel design can offer even more advantages over the model‐based alternative in terms of the proportion of optimal selections. While resulting in similar average numbers of toxicity and efficacy outcomes, the differences in the proportion of correct selections in combination scenarios 7–9 are 3%, 7% and 18%. The moderate improvements correspond to scenarios with increasing efficacy. In scenario 7, WE and Emax select the OBR in 73% and 70% of trials, but WE avoid the selection of inefficacious combination ($d_{A}^{2},d_{B}^{1}$) and highly toxic combination ($d_{A}^{3},d_{B}^{1}$) as by Emax. Moreover, WE selects a dose ($d_{A}^{1},d_{B}^{2}$) with probability nearly 25% which corresponds to three times less toxicity probability, but just a minor drop in efficacy compared to the optimal one. This leads that one of these combinations are chosen in nearly all replicated trials. The largest improvement can be seen in scenario 9 with a plateau in a combination–efficacy relationship. In this case, both designs recommend either of one combination with probability 90%, but the proposed design clearly favours the combination with lower toxicity which results in exposing fewer patients to more toxic combination with the same efficacy. Furthermore, the proposed design results in a higher average efficacy response in this scenario. At the same time, it is important to mention that the benchmark identifies the OBR almost in all simulated trials in all scenarios. While the ratio of proportion of correct selection is relatively high in scenarios 7–9, the lowest ratio is found, again, in the plateau scenario 9 for both of considered methods. This is a consequence that neither the proposed approach or the Emax model are able to model a plateau in the combination–toxicity relationship and still choose the combination with (slightly) higher toxicity.

Overall, the proposed flexible design offers a large gain in the proportion of optimal selections in both single‐agent and combination context with dose/combination–efficacy relationship having a nonmonotonic shape. As expected, the advantages of the proposed design are more substantial in a more advanced setting of the combination trial where one can benefit from not employing any monotonicity or parametric assumptions about the regimen–toxicity and regimen–efficacy relationship. The proposed design is able to terminate the trial due to safety or futility. The cost for the better performance in nonmonotonic scenarios and for a not employing a model is a higher proportion of toxicity responses in scenarios with a jump in toxicity probabilities compared to the model‐based alternative. However, the fact that the design uses no monotonicity or parametric assumptions does not raise any ethical issues in the rest of scenarios and, instead, can result in a higher average efficacy response.

## 5 SENSITIVITY ANALYSIS

The simulation setting as used by Hirakawa (2012) was investigated above under one particular transformation of the random variable. In this section, we study the performance of the proposed design under different specifications of the scenarios and investigate the robustness of the novel design to the proposed logistic transformation.

### 5.1 Correlation

In trials with several endpoints, the correlation between them is important. In the simulation study above, the toxicity and efficacy outcomes were assumed to have a weak correlation which may not hold in an actual trial. Below, we investigate the robustness of the design to the correlation in toxicity and efficacy under scenarios in Table 1. The scenarios 5 and 6 with no OBR are excluded from the analysis as similar results were obtained due to the same safety and futility constraints. The procedure proposed by Tate (1955) and subsequently employed by Hirakawa (2012) is used to generate correlated binary toxicity and continuous efficacy outcomes. The procedure generates a binary normal vector with unit variances and prespecified correlation coefficient ρ. The generated random variable is then transformed to a binary response by applying the normal cumulative distribution function and a Bernoulli quantile transformation, subsequently, and to a continuous normal response by applying the normal cumulative distribution function and a normal quantile transformation.

The proportion of optimal selections in seven scenarios using weak and high negative (r=−0.2,r=−0.8) and positive (r=0.2,r=0.8) correlation structure between toxicity and efficacy outcomes is given in Table 4.

**Table 4:** Proportions of optimal selections in scenarios 1–4 and 7–9 by the proposed WE design for different values of the correlation coefficient

Correlation	Sc 1	Sc 2	Sc 3	Sc 4	Sc 7	Sc 8	Sc 9
r=−0.8	82.8	93.7	90.2	**77.4**	71.2	84.6	60.9
r=−0.2	81.5	94.5	90.8	80.5	72.0	85.6	61.6
r=0.2	82.0	96.9	91.3	81.7	73.1	85.2	62.1
r=0.8	81.1	96.2	91.0	**84.0**	74.8	85.7	63.0

The largest differences are in bold. Results are based on 10^4^ replicated trials.

Overall, the proposed design is robust to the correlation structure of the responses—the difference in the proportion of optimal selection do not differ by both than 3% comparing the high negative and high positive correlation cases in scenarios 1–3 and 7–9. The only exception is scenario 4 in which the difference in the proportion of optimal selection between case r=−0.8 and r=0.8 is equal to 7%. In the case of positive correlation, the selection is shifted toward the beginning of the doses as it becomes more likely to observe a higher efficacy response (corresponding to worse performance) for more toxic doses. In contrast, in case of negative correlation, the selection is shifted further from the beginning of the plateau. These difference can be observed in one scenario only due to the plateau in the dose–efficacy relationship. There are no noticeable changes in other scenarios as the bias caused by the correlation is smaller than the difference in true toxicity and efficacy estimates. Moreover, this does not cause any major changes in the plateau scenario 9 due to a twice larger sample size compared to scenario 4.

### 5.2 Parameters of transformation

The novel design employs the logistic transformation to scale the continuous outcomes to (0,1) interval to be used in the trade‐off function. This logistic transformation, however, uses the information on the minimum and maximum clinically feasible values of the continuous endpoint. While in the context of the motivating study, clinicians are confident in the minimum values ψ=0, the maximum value is more challenging to choose.

The performance of the proposed design above is investigated for the maximum clinically possible value $\psi^{\prime }$. In this section, we investigate the robustness of the design if the upper bound for the continuous endpoint was specified differently. Particularly, we consider the case of a narrower interval of responses $\psi^{\prime }=-3.5$ and wider $\psi^{\prime }=-6.0$ and $\psi^{\prime }=-8.0$. The proportion of optimal selections under different logistic transformation is given in Table 5.

**Table 5:** Proportions of optimal selections in scenarios 1–4 and 7–9 by the proposed WE design for different values of the maximum clinically feasible outcome $\psi^{\prime }$ of the continuous endpoint

Correlation	Sc 1	Sc 2	Sc 3	Sc 4	Sc 7	Sc 8	Sc 9
$\psi^{\prime }=-3.5$	82.5	96.6	**88.7**	81.5	71.5	85.2	64.3
$\psi^{\prime }=-4.5$	82.0	96.9	91.3	81.7	73.1	85.2	62.1
$\psi^{\prime }=-6.0$	80.7	97.0	92.3	82.0	72.6	84.3	61.6
$\psi^{\prime }=-8.0$	80.1	96.9	**92.9**	84.1	71.6	83.8	62.9

The largest differences are in bold. Results are based on 10^4^ replicated trials.

The design is robust to the parameters of the logistic transformation in a reasonable range in the majority of scenarios—the difference in the case of the narrowest $\left(\psi^{\prime }=-3.5\right)$ and widest $\left(\psi^{\prime }=-8.0\right)$ cases does not exceed 2%. Minor improvements can be found for a narrow interval in scenario 1 where the true efficacy parameter for only two safe doses are 0.5 and −0.5. Therefore, having a more steep transformation in the beginning is beneficial. At the same time, narrow interval leads to worsening the performance in scenario 3 where two most efficacious doses have true efficacy parameter −1.5 and −3.0. For the narrow interval, it becomes more difficult for the design to distinguish between these two doses. However, even a slight increase in $\psi^{\prime }$ solves the problem. Importantly, as the bound $\psi^{\prime }$ increases further in a clinically feasible range, the novel design remains to be robust.

### 5.3 Delayed and missing efficacy

In the original setting, it is assumed that the efficacy response is observed together with the toxicity response and regardless whether a patient experienced an adverse event or not. However, in practice, this is unlikely to be true. While toxicity is usually quickly ascertainable, the efficacy endpoint may take longer to be observed (Riviere, Yuan, Jourdan, Dubois & Zohar, 2016). At the same time, waiting for both endpoints increases the length of a trial substantially. Furthermore, in many clinical trials if a patient has experienced an adverse event she will be treated off the protocol and the efficacy outcome cannot be observed. This clearly decreases the total amount of the information obtained in the trial and can influence the outcome of the trial.

The proposed design can be still applied if the efficacy outcome is delayed and/or is missing. In this case, the estimator of the toxicity probability ${\hat{p}}_{t,j}$ and of the mean efficacy response ${\hat{\mu }}_{j}$ is computed based on a different number of observations due to the independence assumption. Consequently, the design can proceed before the full response is observed. We investigate the performance of the novel design under the assumption that it takes twice longer to evaluate an efficacy than a toxicity. We would refer to this simulation setting as “Delayed.” Moreover, we will also assume that the efficacy can be evaluated only in patients with no toxicity response. This setting is referred to as “Missing.” We will also study how these two practical limitations together affect the operating characteristics of the design (“Delayed and Missing”).

The proportions of optimal selections under different practical limitations are given in Table 6.

**Table 6:** Proportions of optimal selections in scenarios 1–4 and 7–9 by the proposed WE design for settings with delayed and missing efficacy outcomes

Correlation	Sc 1	Sc 2	Sc 3	Sc 4	Sc 7	Sc 8	Sc 9
No delayed and no missing	**82.0**	96.9	91.3	81.7	**73.1**	85.2	62.1
No delayed and missing	79.8	96.7	90.5	83.9	70.5	85.7	62.5
Delayed and no missing	80.1	96.6	88.7	82.9	71.7	84.4	63.8
Delayed and missing	**78.9**	96.8	88.9	83.9	**70.0**	84.8	62.7

The largest differences are in bold. Results are based on 10^4^ replicated trials.

Overall, the design is robust to both missing and delayed efficacy response: the difference in the proportion of optimal selection does not exceed 3% compared to the setting with quickly evaluated and no missing responses. As expected, the considered practical limitations lead to a slight decrease in the performance in almost all scenario with scenario 4 being an exception. Under this scenario with a plateau in a dose–efficacy relationship, the missing efficacy conditional on toxicity leads to an increase in the proportion of correct selections by 2%. The missing response shifts the selection towards the beginning of the plateau as further safe dose $\left(d_{A}^{3}\right)$ has three times higher toxicity probability compared to the optimal dose which increases a probability of nonobserving corresponding efficacy responses. Consequently, given the specified prior the design is more confident in the dose $\left(d_{A}^{2}\right)$ to be the optimal one. Finally, we would like to empathize that if an efficacy outcome is available earlier, it should be included in the estimators as it can improve the performance of the design (Mozgunov & Jaki, 2019).

## 6 CONCLUSION

In this work, an extension of the flexible Phase I/II design by Mozgunov and Jaki (2019) for the case of the binary toxicity and continuous efficacy endpoint is proposed. The core idea is to scale appropriate a continuous response to (0,1) and apply it to the information‐theoretic trade‐off function. It is found that the design leads to a substantial gain in the proportion of correct selections in trials involving agent with nonmonotonic dose–efficacy relationships. Furthermore, the design leads to larger improvements compare to the model‐based alternative in advanced combination clinical trials. As the design uses no monotonicity or parametric assumptions about the regimen–toxicity and regimen–efficacy relationship, it can be applied to various complex clinical trials in which fitting a curve can be challenging. The cost of not using a parametric model is an increase (compared to model‐based methods) in the proportion of toxic responses in a scenario with large “jumps” in toxicity probabilities. At the same time, this proportion was found to be controlled below the maximum acceptable toxicity level due to the imposed safety constraint. Finally, the design was found to be robust to the correlation between toxicity and efficacy responses and to the form of the transformation. It also leads to good operating characteristics under the practical limitation such as delayed and missing responses. It is important to mention that there are settings in which borrowing of information between regimen can be used. If this information is available, it can (and should) be used in the design. For example, Mozgunov and Jaki (2018a) considered the special case of the escalation criterion (1) to be used in the CRM design when the monotonicity assumption of the dose–toxicity relationship is satisfied.

**Figure 3:**
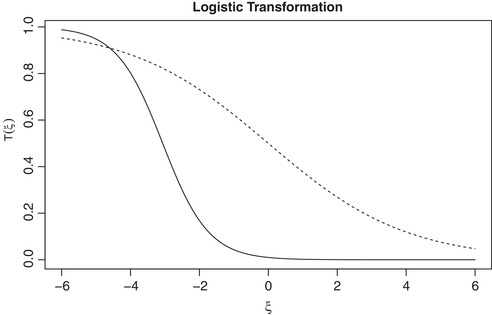
The logistic transformation using the lowest efficacy bound $p_{e}=0.01$ (solid line) and the lowest efficacy bound $p_{e}=0.50$ (dashed line)

The performance of the proposed design depends on the choice of parameters of the logistic transformation, α and β. To derive these parameters, lower and upper efficacy bounds on the continuous scale were mapped to lower and upper bounds on the unit interval. This choice, however, is arbitrary to some extent. An alternative that has been suggested by one of the reviewers is to map the lowest continuous efficacy bound to the probability of efficacy $p_{e}=0.5$ rather than a very low value $p_{e}=0.01$. While the construction of the design and the regimen selection algorithm themselves remains unchanged, one can expect different operating characteristics of the design. Figure 3 below provides the graphs of logistic transformations with the original parameters that used the lowest probability bound $p_{e}=0.01$ and with the lowest probability bound $p_{e}=0.50$ (all other things being equal as in the original proposal). Using the lowest efficacy bound $p_{e}=0.50$, the function (5) is expected to distinguish the regimens better (for the fixed toxicity), for example, if their corresponding means are in the interval (−2,0). While the parameters of transformation studied in the manuscript distinguishes better the regimens, for example, having continuous efficacy in the interval (−2,0) from the regimens having the efficacy in the interval (−4,−3). Therefore, the choice of the lower and upper bounds should depend on the context of a clinical trial. Finally, one can include the choice of these parameters as a calibration step and find the set of parameters that will correspond, for example, to the greatest differences in the selection criterion (5) under the set of clinically feasible scenarios.

The escalation criterion given in Equation (1) can be considered as a loss function (inverse of the utility function), and, in this sense, the approach described in this manuscript is utility‐based. If, however, the target combination is defined in terms of another utility function, one can add more flexibility to the escalation criterion, so it can be tuned such that its minimum value correspond to the maximum value of the specified utility function under the scenarios of interest. This can be achieved by two means. First, one can introduce weights into the equation (1) as

$$\delta \left(\theta ,\gamma \right):={\left(\frac{\gamma_{1}^{2}}{\theta_{1}}\right)}^{\omega_{1}}+{\left(\frac{\gamma_{2}^{2}}{\theta_{2}}\right)}^{\omega_{2}}+{\left(\frac{{\left(1-\gamma_{1}-\gamma_{2}\right)}^{2}}{1-\theta_{1}-\theta_{2}}\right)}^{3-\omega_{1}-\omega_{2}}-1,$$

where each weight $0\leq \omega_{i}\leq 1$ tunes the contribution of the each term. Second, one can alter the parameter of the logistic transformation used to benchmark the continuous efficacy to the unit interval as it is discussed in the previous paragraph.

While a noticeable improvement over the model‐based alternative was found, the benchmark by Mozgunov, Jaki and Paoletti (2018b) revealed that there is still a further room for improvement in scenarios with a plateau in dose–efficacy relationships. A formal testing for the plateau in the relationship incorporated in the WE design can provide means to increase the accuracy in such scenarios and is to be studied further. Furthermore, the setting of the delayed efficacy response was considered assuming that the due to the disease‐specific characteristics of the trial it takes twice longer to evaluate the efficacy and no information is available earlier. At the same, there are clinical trials in which auxiliary information about efficacy is available (e.g., through a short‐term endpoint). Accounting for this information (if available) is crucial and particularly of interest in the setting with no monotonicity assumption. The further of a flexible design accounting for auxiliary information is a subject for further research.

## CONFLICT OF INTEREST

The authors have declared no conflict of interest.

## Supporting information

Supporting Information

Supporting Information
