# Supplementary material for: A flexible design for advanced Phase I/II clinical trials with continuous efficacy endpoints
Source: Biom J. 2019 Jul 12;61(6):1477–92. doi: 10.1002/bimj.201800313 (PMC6899762; doi:10.1002/bimj.201800313)
Supplement: Supplementary file 2 — Supporting Information [file BIMJ-61-1477-s001.pdf]

Biometrical Journal **xx** (xxx) xx, zzz–zzz / DOI: 10.1002/bimj.200100000

A Flexible Design for Advanced Phase I/II Clinical Trials with Continuous Efficacy Endpoints. Supplementary Materials

Pavel Mozgunov[*,1] and **Thomas Jaki** [1]

[1] Medical and Pharmaceutical Statistics Research Unit, Department of Mathematics and Statistics, Lancaster University, Lancaster, LA1 4YF, UK

Received zzz, revised zzz, accepted zzz

Key words: Combination Trial; Continuous Endpoint; Phase I/II Clinical Trial; Non-Monotonic Efficacy.

Comparison of different transformation

While the logistic transformation only was investigated in the main text, alternative mappings might be also plausible. Below, we consider the cumulative distribution function of a standard normal variable (Φ-link) and inverse complementary log-log transformations applied to the single-agent scenarios.

We use the alternatives incorporated in the proposed WE designs using the same parameters as were found for the logistic transformation $\alpha = -4.6$ and $\beta = -1.5$. The logistic, Φ-link and inverse complementary log-log transformations for these choice of parameters are given in Figure 1. We then use them for the WE design.

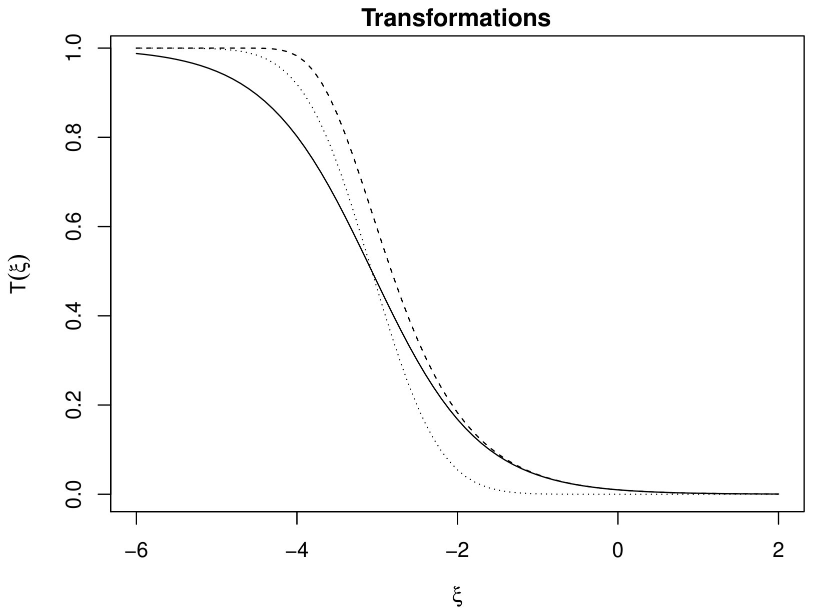

Figure 1 Different transformations for $\alpha^* = -4.6, \beta^* = -1.5$: the logistic (solid line), the Φ-link (dotted line) and the inverse-complementary log-log (dashed line).

*Corresponding author: e-mail: p.mozgunov@lancaster.ac.uk

© xxx WILEY-VCH Verlag GmbH & Co. KGaA, Weinheim

The prior parameters for the designs are kept as in the main text for all transformations and the only different is the link between the outcome of interest ξ and the value on the unit interval to be used in the trade-off function.

The proportions of each regimen selections, proportion of toxicity responses, and mean toxicity responses by the proposed WE design using different transformation is given in Table 1.

Table 1 Operating charactesritscs of the proposed WE design using the logistic, the inverse-probit and the inverse complementary log-log transformation . The columns 'Termination', 'Toxicity' 'Efficacy' correpond to the proportion of earlier termination by each design, the proportion of average toxicity response and the average efficacy response, respectively. Proportion of the OBR are in bold. Results are based on 10^4 replicated trials.

Design	d_A^1	d_A^2	d_A^3	d_A^4	Termination	Toxicity	Efficacy
			Scenario 1				
Logistic	1.5	**82.0**	6.2	0.1	10.3	20.9	−0.6
Φ-link	1.6	**83.9**	3.8	0.0	10.8	25.9	−0.9
Inv. Comp. Log-Log	1.4	**81.7**	6.7	0.0	10.2	20.9	−0.6
			Scenario 2				
Logistic	**96.9**	1.9	0.1	0.0	1.1	20.0	−0.5
Φ-link	**96.4**	2.0	0.0	0.0	1.5	24.6	−0.6
Inv. Comp. Log-Log	**96.6**	2.1	0.2	0.0	1.1	20.1	−0.5
			Scenario 3				
Logistic	0.0	1.0	7.6	**91.3**	0.2	6.3	−2.2
Φ-link	0.0	0.0	2.5	**97.4**	0.0	7.9	−3.0
Inv. Comp. Log-Log	0.0	1.0	7.8	**91.0**	0.2	6.3	−2.2
			Scenario 4				
Logistic	0.0	**81.7**	15.6	0.0	2.6	15.8	−1.7
Φ-link	0.1	**78.5**	18.5	0.0	2.8	21.7	−1.9
Inv. Comp. Log-Log	0.1	**81.1**	16.2	0.1	2.6	15.7	−1.7
			Scenario 5				
Logistic	0.0	0.0	0.0	0.0	**100.0**	8.8	2.0
Φ-link	0	0	0	0	**100.0**	9.5	2.0
Inv. Comp. Log-Log	0.0	0.0	0.0	0.0	**100.0**	8.9	2.0
			Scenario 6				
Logistic	0.5	0.0	0.0	0.0	**99.5**	52.2	−0.1
Φ-link	1.1	0.0	0.0	0.0	**98.8**	60.1	−0.3
Inv. Comp. Log-Log	0.5	0.0	0.0	0.0	**99.5**	52.3	−0.1

Comparing the proportion of correct selections, the WE design using logistic and inverse complementary log-log transformations perform comparably in all considered scenarios with the differences within 0.6%. They also perform similar in terms of proportion of terminations, an average number of toxic and efficacy outcomes. At the same time, the Φ-link leads to a more aggressive allocation of patients. This results in improving the proportion of correct selections under scenario 3 with the OBR being the last dose by nearly 6%, but also in the worsen performance under the plateau scenario 4 by approximately 3%. Importantly, Φ-link also results in an increased average number of toxic responses in all scenarios with the least difference under scenario 3 (7.9 against 6.3 by the logistic transformation) and the greatest difference under scenario 4 (21.7 against 15.8 by the logistic transformation). At the same time, a less conservative allocation leads to an increased average efficacy response under all considered scenarios with the OBR which clearly illustrates the trade-off in the choice of the transformation.

© xxx WILEY-VCH Verlag GmbH & Co. KGaA, Weinheim

Importantly, the transformation above uses the same parameters α and β. However, one can find the values of these parameters such that the proposed designs with various transformations will lead to similar operating characteristics.

Sensitivity of the design to prior parameters

In the many body of the manuscript, the prior calibration details before the safety and futility constraints are imposed were given. Below, we consider the sensitivity of the proportion of correct selections to various steps between toxicity and efficacy mean prior estimates under four scenarios. The results are given in Figure 2.

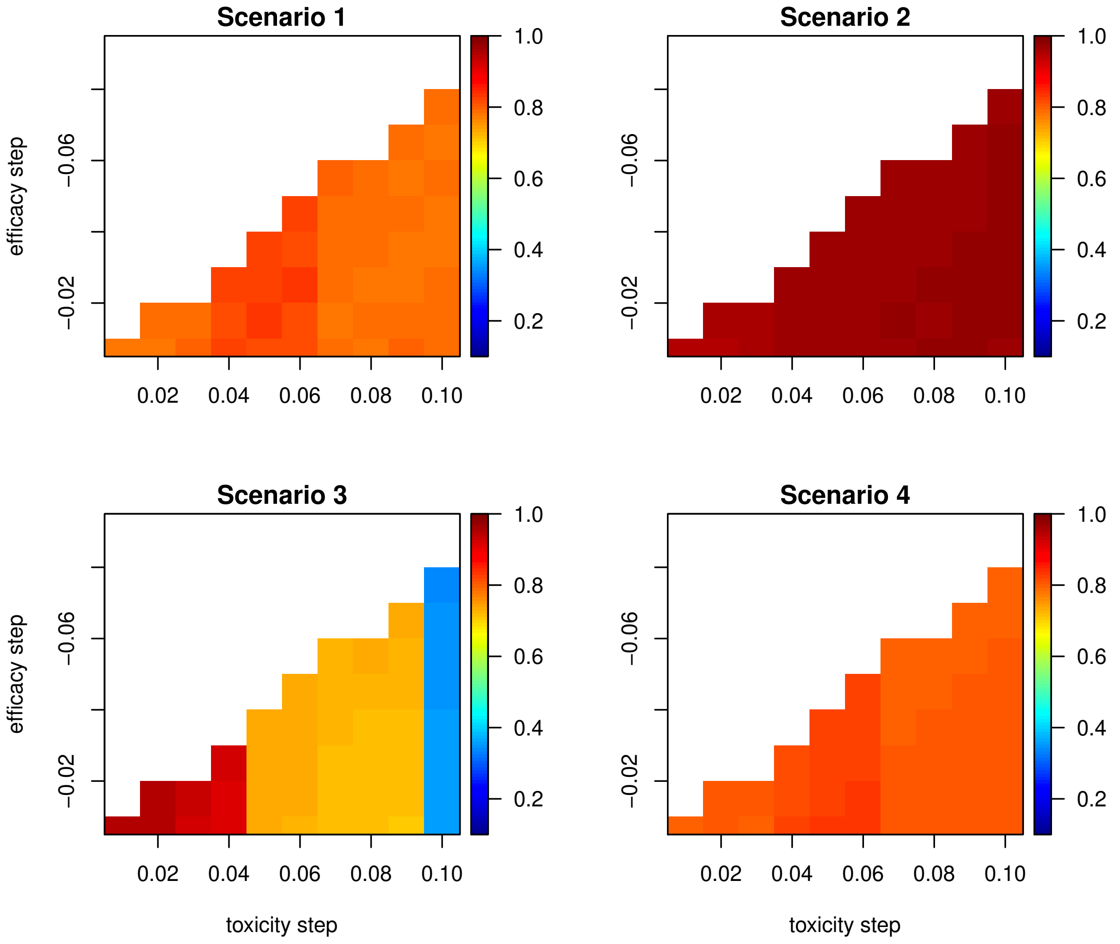

Figure 2 Proportion of OBR selections under different combinations of toxicity and efficacy steps with safety and futility constraint imposed.

There is a wide range of values of prior parameters, which lead to a high proportion of correct selections under all four scenarios, and the design is robust to the choice of the prior distributions parameters. An only point of concern, however, is a noticeable worsen performance under scenario 3 for the toxicity step of 0.10. This drop in the proportion of selections is due to the calibrated time-varying safety constraint. The chosen values make it is less likely that the design will reach regimen that are higher in the initial

© xxx WILEY-VCH Verlag GmbH & Co. KGaA, Weinheim

ordering. Nevertheless, it worth pointing out that the performance is within of 10% difference for toxicity steps between 0.01 and 0.09.

© xxx WILEY-VCH Verlag GmbH & Co. KGaA, Weinheim
